# Case Report: Prolonged VV-ECMO (111 Days) Support in a Patient With Severe COVID-19

**DOI:** 10.3389/fmed.2021.681548

**Published:** 2021-08-05

**Authors:** Zhiheng Xu, Yonghao Xu, Dongdong Liu, Xuesong Liu, Liang Zhou, Yongbo Huang, Yimin Li, Xiaoqing Liu

**Affiliations:** Department of Critical Care Medicine, State Key Laboratory of Respiratory Diseases, Guangzhou Institute of Respiratory Health, First Affiliated Hospital of Guangzhou Medical University, Guangzhou Medical University, Guangzhou, China

**Keywords:** COVID-19, acute respiratory failure, acute respiratory disease syndrome, extracorporeal membrane oxygenation, prolonged maintenance

## Abstract

Venovenous extracorporeal membrane oxygenation (VV-ECMO) may be a lifesaving rescue therapy for patients with severe coronavirus disease 2019 (COVID-19). However, little is known regarding the efficacy of prolonged ECMO (duration longer than 14 days) in patients with COVID-19. In this case report, we report the successful use of prolonged VV-ECMO (111 days) in a 61-year-old man with severe COVID-19. Given the high mortality rate of severe COVID-19, this case provided evidence for use of prolonged VV-ECMO as supportive care in patients with severe COVID-19.

## Introduction

As of January 3, 2021, 84,793,806 infections by severe acute respiratory syndrome coronavirus-2 (SARS-CoV-2) and 1,838,440 related deaths had occurred worldwide (1). The mortality rates of patients with severe coronavirus disease 2019 (COVID-19) admitted to intensive care units (ICUs) is high (2–4). Venovenous extracorporeal membrane oxygenation (VV-ECMO) may serve as a lifesaving rescue therapy (5, 6). A recent report from 213 hospitals worldwide based on Extracorporeal Life Support Organization (ELSO) registry data provided a generalizable estimate of about 40% ECMO mortality in patients with severe COVID-19 (7). The median duration of ECMO support was 13.9 days (7). The data supported current recommendations that centers experienced in ECMO should consider its use for refractory COVID-19-related respiratory failure (7, 8). However, little is available regarding prolonged VV-ECMO (duration longer than 14 days) in patients with COVID-19. Here, we report the case of a patient with severe COVID-19 who received prolonged VV ECMO and was successfully decannulated after 111 days.

## Case Report

A 61-year-old man with a height of 168 cm and a predicted body weight of 64.2 kg had recently returned from Wuhan. He presented with fever (38.5°C), dry cough, and hypodynamia. Nasopharyngeal swabs obtained at the time of presentation were positive by PCR for SARS-CoV-2. A chest computed tomography (CT) scan demonstrated bilateral air-space infiltrates with consolidation and ground glass opacities consistent with a diagnosis of COVID-19. He had a medical history of sleep apnea hypopnea syndrome, hypertension, and chronic hepatitis B.

The patient's respiratory status deteriorated on Day 10 post-hospitalization, requiring intubation. He was transferred to the ICU of the First Affiliated Hospital of Guangzhou Medical University, the designated center for patients with COVID-19 in Guangdong, China.

## Initiation of ECMO

**Figure 1:**
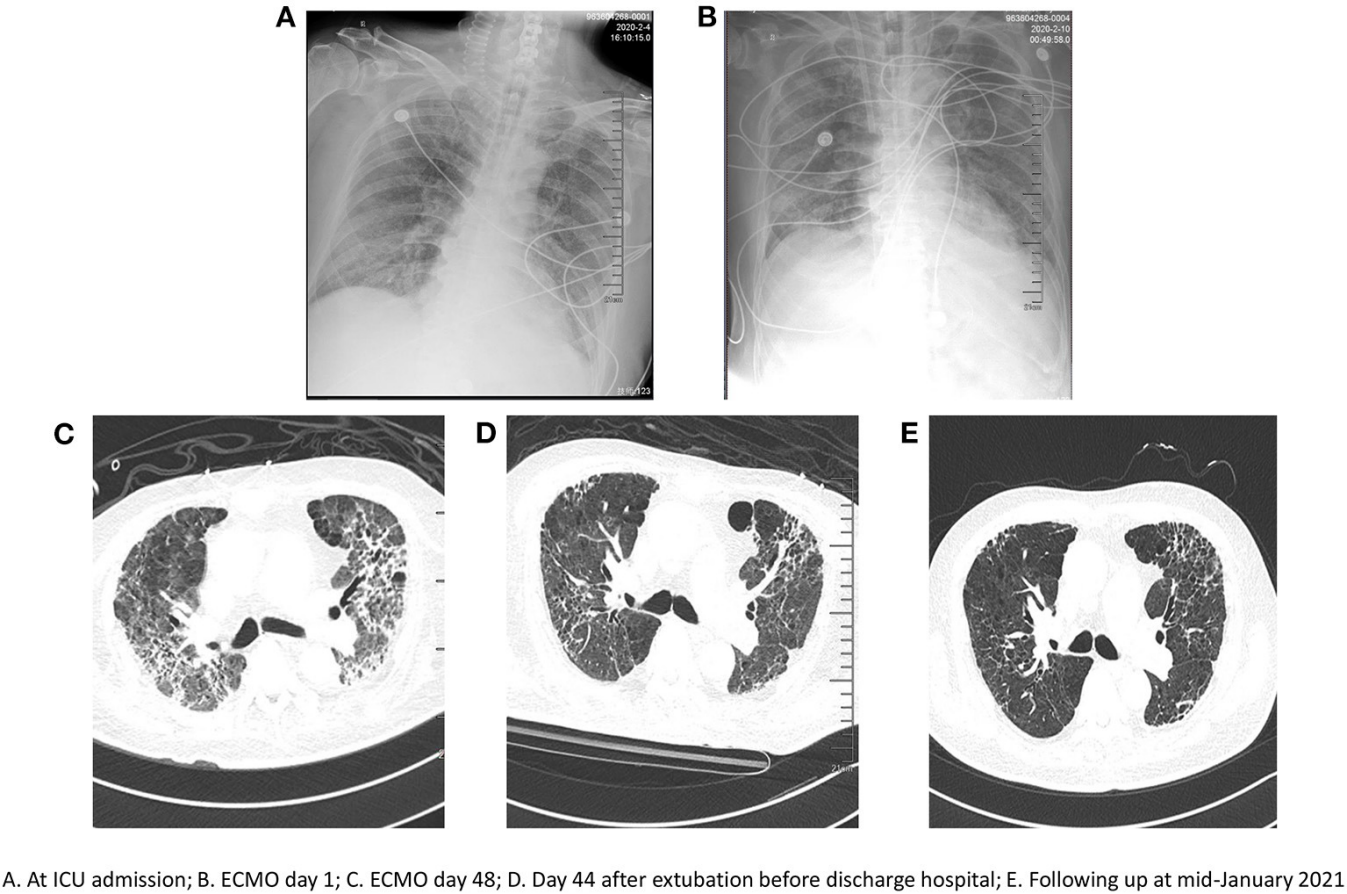
Serial chest radiographs. **(A)** at ICU admission; **(B)** ECMO day 1; **(C)** ECMO day 48; **(D)** Day 44 after extubation before discharge hospital; **(E)** Following up at mid-January 2021.

On Day 6 post-intubation, the patient's oxygenation level continued to deteriorate despite lung-protective ventilation, high positive end-expiratory pressure (PEEP), deep sedation, and paralysis using neuromuscular blockers. His PaO_2_/FiO_2_ ratio decreased from 200 to 123 mmHg and his PaCO_2_ increased from 50 to 64 mmHg. Chest x-ray revealed diffuse opacification in the lung field (Figures 1A,B). A decision was made to commence VV-ECMO on Day 6 post-intubation for respiratory failure.

Cannulation was carried out *via* a right femoral–right internal jugular vein approach. Initial ECMO settings were sweep gas flow 1.5 L/min, flow 4.4 L/min, 3,005 RPM, and heparin infusion [with the goal of achieving an activated partial thrombosis time (APTT) of 50–60 s]. Ventilation was set at a tidal volume of 6 ml/kg, PEEP 10 cmH_2_O, and a respiratory rate of 20 bpm to keep his plateau pressure <25 cmH_2_O.

Chest CT did not show any significant recovery and compliance remained poor over several weeks following initiation of ECMO (Figure 1C). Infectious complications included Gram-positive and Gram-negative bacteria and fungi (e.g., *Enterococcus faecium, Escherichia coli*, or *Candida albicans*) based on bronchoscopic alveolar lavage and sputum cultures. Antibiotics were administered as required based on the laboratory data from bacterial cultures.

### Anticoagulation and ECMO Circuit Change

**Figure 2:**
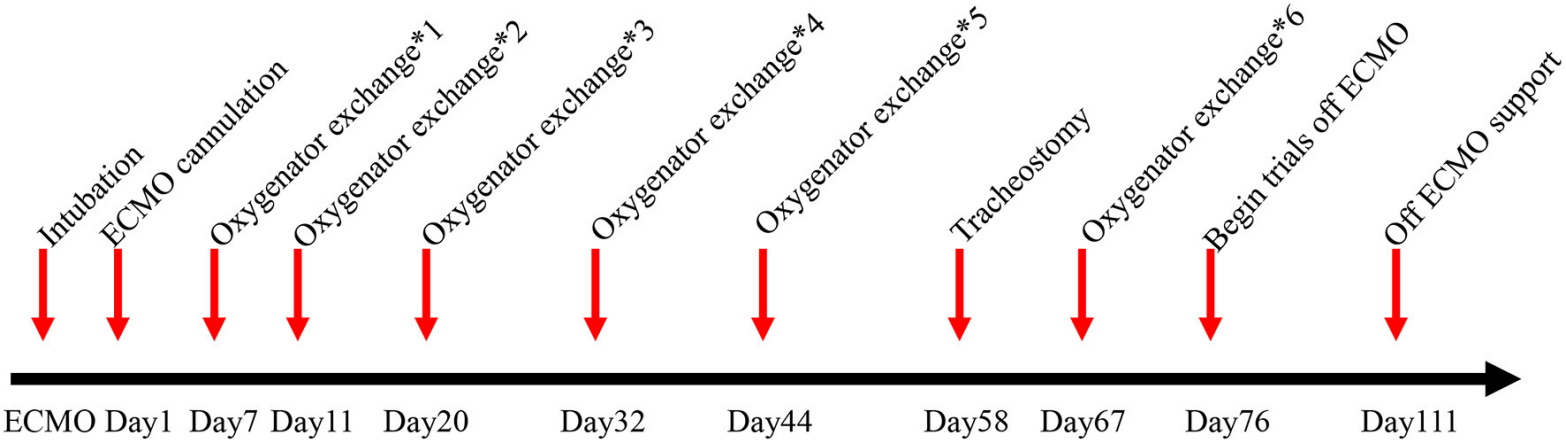
Timeline of ECMO events.

Coagulation function was reviewed every 3 h after initiation of ECMO and heparin infusion. Heparin (starting at 1 μg/kg/h) was administered and the dose titrated to achieve an APTT ranging from 50 to 60 s. Both bleeding and thrombus formation were monitored to adjust the dosage of heparin. However, active surveillance for hemolysis revealed elevated D-dimer on Day 7 after VV-ECMO initiation. Minimal areas of thrombus formation were observed peripherally on the oxygenator membrane. Meanwhile, the patient was in respiratory distress and laboratory tests showed clear evidence of deterioration of oxygenation. This was resolved following an oxygenator change out and his SpO_2_ and PaO_2_/FiO_2_ ratio improved. However, 4 days after oxygenator change out (ECMO Day 11), his PaCO_2_ increased and thrombi appeared on the membrane again, again requiring oxygenator change out. In the following days, the membrane oxygenator was changed out four times because of gas exchange failure, apparent thrombus formation on the membrane, and acute D-dimer increases associated with massive clot formation around the hollow-fiber bundles in the oxygenator (Table 1; Figure 2). The average lifespan of the oxygenators was 11 days.

**Table 1:** Timeline of medical and ECMO events in a patient with COVID-19.

**Event**	**Date**	**ECMO Day**	**Comments**
Symptoms began	Jan. 16, 2020	–	–
Hospital admission	Jan. 22, 2020	–	PCR (+)
ICU admission	Jan. 23, 2020	–	Dyspnea
Intubation	Feb. 3, 2020	–	Respiratory failure
Transfer to referring hospital	Feb. 4, 2020	–	–
ECMO cannulation	Feb. 9, 2020	1	Oxygenation and hypercapnia continued to deteriorate
Oxygenator exchange*1	Feb. 15, 2020	7	Oxygenation deteriorate and high D-dimer
Oxygenator exchange*2	Feb. 19, 2020	11	Thrombus on oxygenator membrane and high PaCO_2_
Oxygenator exchange*3	Feb. 28, 2020	20	Low oxygenation, high PaCO_2_ and increased D-dimer
Oxygenator exchange*4	Mar. 12, 2020	32	Bleeding, low fibrinogen, and increased D-dimer
Oxygenator exchange*5	Mar. 24, 2020	44	Oxygenation deteriorate and thrombus on oxygenator membrane
Tracheostomy	Apr. 7, 2020	58	–
Orotracheal intubation	Apr. 10, 2020	61	Bleeding of tracheal incision and rhinal nasal cavity
Begin trials off ECMO*1	Apr. 15, 2020	66	Failure of low oxygenation and high PaCO_2_
Oxygenator exchange*6	Apr. 16, 2020	67	Bleeding and thrombus on oxygenator membrane
Begin trials off ECMO*2	Apr. 25, 2020	76	Gradually decreased analgesia and sedation
Off ECMO support	May. 29, 2020	111	–
Extubation	Jul. 2, 2020	–	Successful Spontaneous Breathing Test on Jun. 30, 2020
Discharge hospital	Aug. 27, 2020	–	–

A tracheostomy was performed on ECMO Day 58. However, bleeding from the tracheal incision and nasal cavity was hard to control under ECMO support with heparin anticoagulation. The dose of heparin had to be decreased, and thrombi again appeared on the membrane. Bleeding of the tracheal incision and nasal cavity was finally controlled using tranexamic acid. Then, the dosage of heparin could be increased, and the infusion of blood products (such as fresh frozen plasma, fibrinogen, and platelets) could be reduced to avoid thrombus formation. We first began trials off ECMO after 66 days of support. The patient again showed a low PaO_2_/FiO_2_ ratio and a high PaCO_2_ with decreased ECMO support. A sixth oxygenator exchange had to be performed.

### Subsequent Progress

The patient's condition slowly improved when balance was achieved in terms of bleeding, thrombus formation, and fluids. With the appropriate use of antibiotics, analgesia and sedation were gradually reduced. Moreover, there was a major improvement in compliance (Table 2). During this time, the patient was awake, alert, intubated, and on light sedation for comfort. After prolonged VV-ECMO support for 111 days, the patient was successfully decannulated. He underwent daily physical therapy and continued physical therapy with the intubated cannula in place. On July 2, 2020, he was successfully weaned off mechanical ventilation. Significant improvement in his chest CT was observed before discharge (Figure 1D). Finally, on August 27, 2020, he was discharged from hospital and was able to walk slowly by himself after a prolonged hospitalization of 218 days. The patient was followed up every 3 months without readmission. He received home oxygen therapy about 12 h per day. Significant improvement in his chest CT was observed in mid-January 2021 (Figure 1E).

**Table 2:** Ventilation parameters and respiratory mechanics during ECMO.

**Timing**	**FiO_**2**_**	**PaO_**2**_**	**P/F ratio**	**VT**	**RR**	**PEEP**	**ΔP**	**Compliance**
		**(mmHg)**	**(mmHg)**	**(ml)**	**(per/min)**	**(cmH_**2**_O)**	**(cmH_**2**_O)**	**(ml/cmH_**2**_O)**
Before ECMO	0.7	86.6	123	470	21	11	15	25
ECMO day 1	0.5	84.1	168	400	16	11	12	27
Before decannulation	0.6	119	198	530	25	6	16	30
After decannulation	0.6	121	201	530	25	6	16	36

*FiO_2_, fraction of inspired O_2_; PaO_2_, partial pressure of oxygen in arterial blood; P/F ratio, PaO_2_/FiO_2_ ratio; VT, tidal volume; RR, respiratory rate; PEEP, positive end expiratory pressure; ΔP, driving pressure*.

## Discussion

The use of ECMO to treat acute respiratory distress syndrome (ARDS) is currently in widespread use (9). As ECMO management is improving, prolonged duration of support is becoming more common. The ELSO registry data showed that 4,361 adult patients who underwent prolonged ECMO for respiratory failure had a mean ECMO duration of 22 days (10). Moreover, previous cases demonstrated that prolonged ECMO support for 265 days without complications was possible; one patient received ECMO for 403 days while waiting for lung transplantation but died soon after decannulation (11, 12). In our case, VV-ECMO was maintained for 111 days and successful weaning and recovery of lung function was achieved in a patient with severe COVID-19.

Several factors may be associated with the successful use of prolonged VV-ECMO in critically ill patients with COVID-19. First, early use of ECMO is recommended in these patients. In the ECMO to Rescue Lung Injury in Severe ARDS (EOLIA) study, patients with severe ARDS received immediate VV-ECMO if indicated by one of three criteria: a PaO_2_/FiO_2_ ratio <50 mmHg for more than 3 h; a PaO_2_/FiO_2_ ratio <80 mmHg for more than 6 h; or an arterial blood pH <7.25 with PaCO_2_ > 60 mmHg for more than 6 h (13). However, these criteria may be controversial because of several instances of “late” use of ECMO (14). By contrast with the EOLIA criteria, ECMO was administered in this patient at a relatively “early” point when we observed a rapid decline in oxygenation (PaO_2_/FiO_2_ ratio decrease from 200 to 123 mmHg) and an increase in hypercapnia (PaCO_2_ increase from 50 to 64 mmHg).

Second, coagulation function was continuously monitored. Thrombotic complications and coagulopathy frequently occur in patients with COVID-19 (15). In addition, bleeding and thrombosis are serious complications during the use of ECMO (16). Taken together, the data suggest that use of ECMO in patients with COVID-19 may be challenging, particularly if prolonged support is needed. In this case, we continuously monitored coagulation (APTT, D-dimer, fibrinogen, and fibrinogen degradation products) to detect thrombotic bleeding and hemolytic complications. Coagulation disorders were the main reason why a change of the oxygenator is required in this patient. Balancing bleeding with risk of thrombus formation was of vital importance in the care of this patient who was bleeding from a tracheal incision and nasal cavity. Antiplasmin therapy (tranexamic acid) may have been critical to the care of this patient. When bleeding was controlled by tranexamic acid, we were able to increase the dosage of heparin and reduce the infusion of blood products (fresh frozen plasma, fibrinogen, and platelets). Decreased administration of blood product also meant reduced risks of thrombosis formation on the oxygenator. Meanwhile, aggressive ECMO circuit changes may improve membrane oxygenator-related coagulation disorders.

Third, infection control was performed according to the ELSO's guideline on patients with COVID-19 (17). The patient was managed in a negative pressure isolation room. ECMO team members received adequate training in the use of PPE including N95/FFP2 masks, gowns, cap, and eye protectors. Antibiotics were administered as required based on laboratory data from bacterial cultures. Moreover, ours was the designated center for patients with COVID-19 in Guangdong, China, and thus we implemented additional level of infection prevention and control measures such as use of closed respiratory suction tubes and disposable bronchoscopy tubes.

Fourth, centers experienced in ECMO are recommended to deliver ECMO to patients with refractory respiratory failure because this strategy is associated with low mortality (18). The ICU of the First Affiliated Hospital of Guangzhou Medical University was the designated center for patients with severe COVID-19 and experienced with ECMO because of the high volume of cases every year (19). Finally, the relatively small number of patients with COVID-19 in Guangdong Province prompted intensive care interventions, and availability of ICU beds may have contributed to enhanced levels of care for patients like the one described here (20, 21).

## Conclusion

Our patient was aggressively treated by early use of ECMO, and coagulation was continuously monitored to inform the need for circuit change. The patient was ambulatory at the time of discharge despite a prolonged VV-ECMO support of 111 days. Future studies are warranted to determine reversibility of lung injury following use of ECMO in patients with COVID-19.

## Data Availability Statement

The original contributions presented in the study are included in the article/supplementary material, further inquiries can be directed to the corresponding author/s.

## Ethics Statement

The studies involving human participants were reviewed and approved by the Ethics Commission of the First Affiliated Hospital of Guangzhou Medical University. The patients/participants provided their written informed consent to participate in this study.

## Author Contributions

XiL, ZX, YX, and YL: conception and design. YL and XiL: administrative support. YH, DL, and LZ: provision of study materials or patients. ZX, DL, XuL, and YH: collection and assembly of data. ZX, YX, and XuL: manuscript writing. All authors contributed to the article and approved the submitted version.

## Conflict of Interest

The authors declare that the research was conducted in the absence of any commercial or financial relationships that could be construed as a potential conflict of interest.

## Publisher's Note

All claims expressed in this article are solely those of the authors and do not necessarily represent those of their affiliated organizations, or those of the publisher, the editors and the reviewers. Any product that may be evaluated in this article, or claim that may be made by its manufacturer, is not guaranteed or endorsed by the publisher.
